# Testimony of Dr. Henry Holland, of London, Respecting the Character of Medical Schools

**Published:** 1848-10

**Authors:** 


*Testimony of Dr. Henry Holland, of London, respecting the Character of Medical Schools.*

It is mentioned in the Annual Announcement of Jefferson Medical College, of Philadelphia, that "Dr. Henry Holland, one of the most learned and accomplished physicians of London, who visited this country a few years ago," in his evidence given before the "Committee on Registration of the British House of Commons," in answer to a question put to him by Mr. T. B. Macaulay:—"Does not the American Medical school stand high?" replied, "I should rank it next to the English on the whole." The exception in favor of the schools of his own country was a natural and proper tribute of patriotism. We are to understand its significancy to be the same as when an absent person is said to have qualities superior to those of any other, "*the present company always excepted.*"

